# Para Hisian Pacing: Is There More Than One Way to Conduct From The Ventricle Back to The atria?

**Published:** 2011-03-25

**Authors:** Siva K Mulpuru, Subha L Varahan, Navinder S Sawhney

**Affiliations:** Division of Cardiac Electrophysiology, University of California, San Diego

A 72 year old male with past medical history of hypertension and dyslipidemia presented with a 10 year history of palpitations associated with dizziness. His physical exam was within normal limits. His LV function was 62% and left atrial size was within normal limits. He was euthyroid and a recent stress test was negative for inducible ischemia. During the stress test he developed a LBBB morphology tachycardia that converted to narrow complex tachycardia without any appreciable change in rate on the surface EKG. As he had symptomatic recurrences on beta blockers he was brought to the electrophysiology lab for a diagnostic study and possible ablation.

A Para Hisian pacing maneuver was performed at baseline as shown in Figure 1. High out pacing was initiated from the His bundle distal bipole and the output was lowered to capture only the ventricular myocardium and not the His bundle.

Which of the following statements is correct regarding the patient's baseline conduction system?

1. Only nodal response is seen and septal accessory pathway can be ruled out.

2. There is presence of an accessory pathway

3. There may be presence of more than once accessory pathway.

4. The maneuver cannot be interpreted as a retrograde His bundle cannot be seen with loss of capture.

## Answer

Only nodal response is present and septal accessory pathway can be ruled out.

## Explanation

The tracing shown above illustrates a common pitfall encountered during the parahisian pacing maneuver. For the first 3 beats the QRS complexes are narrow suggestive of His bundle capture in addition to ventricular myocardial capture. Stimulus- Atrial electrogram time measured in the CS proximal bipole is 82 msec. The forth beat is wide suggestive of only ventricular myocardium capture.  The stimulus- A time measured is 158msec. Prolongation of stimulus- A time with ventricular myocardium only capture suggests nodal response and rules out a para septal accessory pathway. The fifth beat is also narrow with very short stimulus- A time at 42 msec. This short Stimulus- A time could be mis-interpreted as extranodal response; however it is the result of simultaneous His, atrium and ventricular capture. The sixth beat is due to atrial capture only and illustrates that the bipole can have relative movement in the heart chambers with respiration which can alter what tissue gets captured.

Para Hisian maneuver relies on 3 key facts:

1.The atria and the ventricles are electrically isolated from each other by the fibrous skeleton of the heart except in the region of His bundle.

2.The His bundle is an insulated structure and requires high output current to capture. Local ventricular myocardial capture near the His bundle has to first proceed to the apex to get into the conduction fibers and then climb up the right bundle to reach the His bundle and the AV node.

3.During high output His bundle capture the atria and the ventricle are activated simultaneously. Whereas with local ventricular myocardial capture the signal first has to travel to the RV apex and then the right bundle branch, His bundle, AV node and the atria are activated in series and hence the increase in stimulus to A time.

Para Hisian pacing helps to detect a para septal accessory pathway at baseline and does not rule out free wall pathways or pathways with decremental conduction [1].  One of the caveats of interpreting the Para Hisian pacing maneuver is to ensure that the atrium is not captured by stimulus. While it is good to see His electrogram pulled into the stimulus with His bundle capture, narrowing and widening of QRS in the surface leads can be used as surrogates to assess His bundle capture. Ventricular pacing during complete heart block induced by adenosine infusion didn't demonstrate any retrograde VA conduction ruling out the presence of an accessory pathway. The patient presented in this case had inducible AVNRT and a slow pathway modification rendered him non inducible.

## Figures and Tables

**Figure 1:**
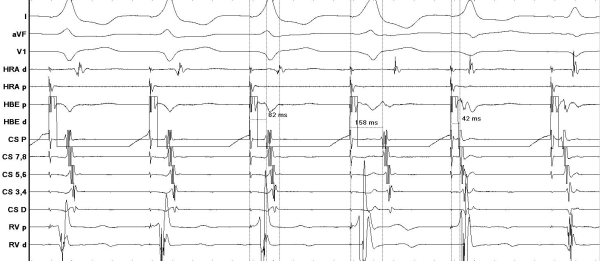
Para Hisian pacing maneuver was performed at base line.  The first 3 beats demonstrate ventricular myocardial capture with His bundle capture with a narrow QRS complex. The 4th beat is wide suggestive of local myocardial capture only and loss of His bundle capture. The 5th beat is also narrow with very short stimulus- A time at 42 msec which is the result of simultaneous His, atrium and ventricular capture. The sixth beat is due to atrial capture only.
